# Design and Synthesis of a Series of Truncated Neplanocin Fleximers

**DOI:** 10.3390/molecules191221200

**Published:** 2014-12-16

**Authors:** Sarah C. Zimmermann, Elizaveta O’Neill, Godwin U. Ebiloma, Lynsey J. M. Wallace, Harry P. De Koning, Katherine L. Seley-Radtke

**Affiliations:** 1 Department of Chemistry & Biochemistry, University of Maryland, Baltimore County, 1000 Hilltop Circle, Baltimore, MD 21250, USA; 2 Institute of Infection, Immunity and Inflammation, College of Medical, Veterinary and Life Sciences, University of Glasgow, 120 University Place, Glasgow G12 8TA, UK

**Keywords:** fleximer, carbocyclic nucleosides, 3-deazaneplanocin A, SAHase, trypanomiasis

## Abstract

In an effort to study the effects of flexibility on enzyme recognition and activity, we have developed several different series of flexible nucleoside analogues in which the purine base is split into its respective imidazole and pyrimidine components. The focus of this particular study was to synthesize the truncated neplanocin A fleximers to investigate their potential anti-protozoan activities by inhibition of S-adenosylhomocysteine hydrolase (SAHase). The three fleximers tested displayed poor anti-trypanocidal activities, with EC_50_ values around 200 μM. Further studies of the corresponding ribose fleximers, most closely related to the natural nucleoside substrates, revealed low affinity for the known *T. brucei* nucleoside transporters P1 and P2, which may be the reason for the lack of trypanocidal activity observed.

## 1. Introduction

Modified nucleosides, in particular carbocyclic nucleosides, are potent inhibitors of S-adenosyl homocysteine hydrolase (SAHase) [1]. SAHase is a critical enzyme that hydrolyzes S-adenosyl homocysteine, the byproduct of biomethylations that utilize S-adenosylmethionine (SAM) [2,3]. By inhibiting SAHase, an excess of SAH is produced, which in turn exhibits potent inhibitory effects on methyltransferases [4]. Thus, inhibition of SAHase leads to incomplete methylation of nucleic acids, phospholipids, proteins, and other small molecules, disrupting various biochemical pathways [5]. As a result, carbocyclic nucleosides have proven useful in a number of chemotherapeutic applications [6,7,8].

**Figure 1:**
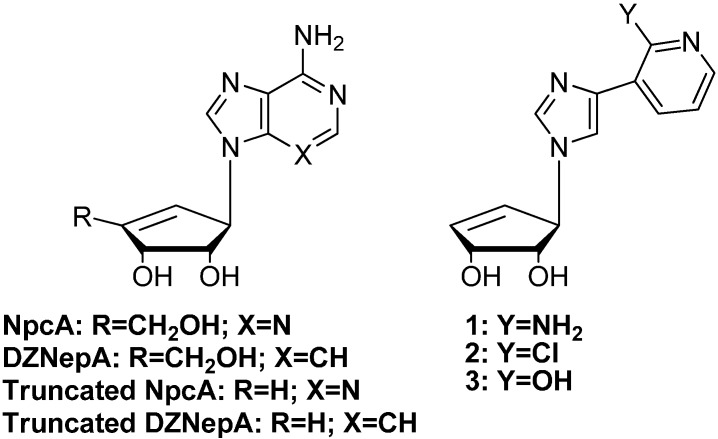
Neplanocin A (NpcA) and analogues and the target NcpA fleximers (**1**–**3**).

Neplanocin A (NpcA, Figure 1) and aristeromycin (Ari) are both naturally occurring carbocyclic adenosine analogues that have shown significant antiviral, antiparasitic and anticancer properties [4,7,9,10]. Unfortunately, NpcA and Ari both exhibit deleterious cytotoxicity due to intracellular conversion to their triphosphate forms by adenosine kinase as well as their recognition and metabolism by adenosine deaminase [11,12,13]. Removal of the 4'-CH_2_OH from Ari and NpcA, as shown in the truncated analogues shown in Figure 1 (R = H), significantly lowers the cytotoxicity [14].

Interestingly, nucleosides with base modifications such as 3-deazaadenosine have also been found to act as substrates, with similar K_m_’s found for adenosine and 3-deazaadenosine [7,10,15]. To date, the truncated 3-deaza analogues of Ari and NpcA (truncated DZNepA, Figure 1) lacking the 4'-hydroxy-methyl group have both exhibited greater levels of inhibition than their parent counterparts [5,6,13,16].

More importantly, these compounds have also shown potent inhibition against chloroquine-resistant and chloroquine-susceptible strains of *P. falciparum* [5]. In protozoan parasites, methylation of the four nucleosides present in the “cap-four” terminal end of mRNA requires SAM as the methyl donor. This cap structure is important for RNA recognition and stability, is highly conserved across almost all protozoan species, and is critical for replication [17,18,19]. Thus, inhibition of SAHase results in an accumulation of SAH, causing methylations to cease, which then disrupts the methylation of the cap structure, thereby providing an important target for the development of potential antiparasitic chemotherapeutics [5].

The Seley-Radtke group has long been interested studying the effects of flexibility on the nucleobase. This flexibility is achieved by “splitting” the purine base into its respective imidazole and pyrimidine (or pyridine) components, which remain connected by a single carbon-carbon bond between the two heteroaromatic moieties. This connectivity allows for free rotation, while still retaining the elements essential for base pairing and molecular recognition [20,21,22,23,24,25,26,27,28]. This modification has led to enhanced enzyme binding and recognition, as well as the ability to overcome point mutations in enzyme binding sites [29,30]. These analogues have also been studied for their potential therapeutic properties [20,21,22,23,24,25,26,27,28]. Interestingly, when the fleximer analogues of adenosine (Flex-A), inosine (Flex-I) and guanosine (Flex-G) were studied in SAHase, which is a flexible enzyme, Flex-A and Flex-I acted as substrates, whereas Flex-G proved to be an inhibitor [22]. This is significant because it is, to our knowledge, the only report of a G-nucleoside inhibiting an adenosine metabolizing enzyme. It has been postulated that this is due to an intramolecular hydrogen bond between the pyrimidine and the 5'-OH of the sugar, which then positions the amino group into the binding site where the amino group on adenosine would normally reside, thus essentially creating an adenosine mimic [22].

Historically, a number of nucleoside analogues have been evaluated for trypanocidal activity [31,32,33,34]. For example, Cai *et al.* showed that the antiviral drug ribavirin was an inhibitor of *Trypanosoma cruzi* SAHase [33]. Additionally, 7-deaza-5'-noraristeromycin was shown to be a potent inhibitor of four strains of *Trypanosoma brucei* [34]. To further explore the potential of base flexibility and antiparasitic activity, we combined the fleximer base with the carbocyclic nucleoside scaffold, to determine whether the flexible base motif would enhance the biological results previously observed with carbocyclic analogues such as NpcA and Ari. Thus, a series of 3-deaza fleximers (compounds **1**–**3**, Figure 1) were designed and synthesized to evaluate their anti-parasitic properties.

## 2. Results and Discussion

### 2.1. Chemistry

**Scheme 1:**
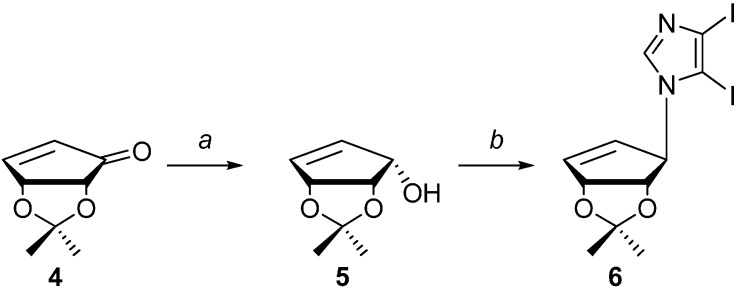
Synthesis of compound **6**.

As shown in Scheme 1, cyclopentenol **5** was available from known literature procedures starting from d-cyclopentenone **4** [35], which can be obtained following stereospecific reduction to the “down” hydroxyl using Luche reduction conditions [36]. Alcohol **5** was then coupled to 4,5-diiodoimidazole [29] using standard Mitsunobu [37] conditions to give **6**. Initially the Mitsunobu reaction was attempted with diisopropylazodicarboxylate (DIAD) and triphenylphosphine (TPP) in dichloromethane at room temperature to yield **5**, however only in a 12% yield. Attempts at heating the reaction only served to give additional side products, as well as to lower the yield even further. Changing the solvent to THF increased the solubility of the diiodoimidazole and subsequently resulted in an improved yield of 40%. Unfortunately, contaminates from the byproduct, triphenylphospine oxide (TPPO), still proved to be problematic during purification. Altering the phosphine reagent to DPPE (1,2-bis(diphenylphosphino)ethane) drastically improved the ease in purification. Other coupling methods were also tried, such as using Hendrickson’s “POP” reagent, bis(triphenyl)oxodiphosphonium trifluoromethanesulfonate [38], or using bases such as NaH or K_2_CO_3_ [39] to form the imidazole nucleophile, proved unsuccessful when compared to the Mitsunobu coupling using DPPE.

*Reagents and Conditions*: *a*. CeCl_3_·7H_2_O, MeOH, NaBH_4_; *b*. DPPE, DIAD, 4,5-diiodoimidazole, THF, rt.

**Scheme 2:**
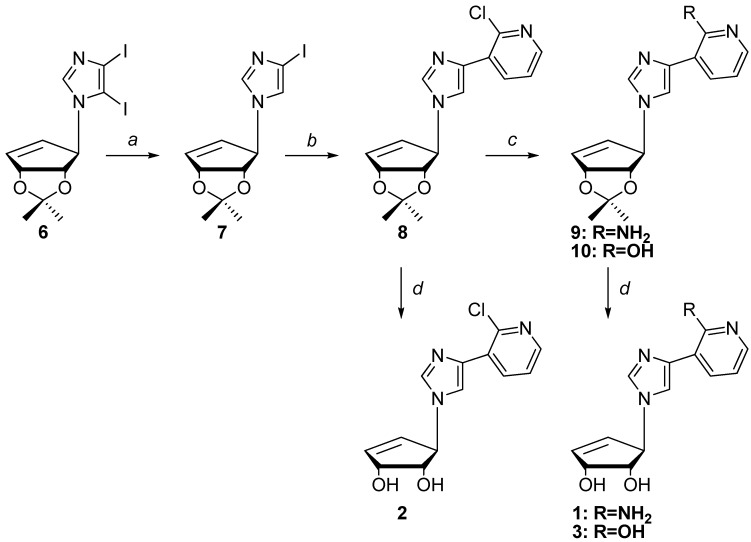
Synthesis of compounds **1**–**3**.

Next, as shown below in Scheme 2, removal of the 5-iodo group of **6** to give compound **7** was achieved via selective deiodination using ethyl magnesium bromide (EtMgBr) followed by quenching with water. Coupling to the pyridine ring was then accomplished using Stille [40] coupling.

*Reagents and Conditions*: *a*. EtMgBr, THF, 0 °C; *b*. 3-tributyltin-2-chloropyridine, Pd(PPh_3_)_4_, Cu(I) Br, 1,4-dioxane, reflux; *c.* for compound **9**: (i) hydrazine neat, 80 °C; (ii) TiCl_3_; for compound **10**: concentrated acetic acid neat, 110 °C; *d.* TFA/H_2_O (1/1) in THF.

The 3-tributyltin-2-chloropyridine was prepared from the commercially available 3-bromo-2-chloropyridine. Stille coupling of **7** with the 3-tributyltin-2-chloropyridine provided **8** in a 23% yield, however when copper (I) bromide was used, the yield improved to 71%. Following Stille coupling, transformation of the chloro group into the exocyclic amine group was necessary. Standard procedures using MeOH/NH_3_ or converting the chloro to an azide using sodium or lithium azide proved unsuccessful. A literature search revealed a palladium-assisted method developed by Hartwig using sodium *t*-butoxide in ammonia saturated 1,4-dioxane [41]. Unfortunately this method also proved unsuccessful.

Related to this latter route, Buchwald developed a similar method, where the catalyst is made *in situ* using a more common phosphine ligand [42]. This method seemed promising since one of the examples utilized 2-chloropyridine, which was successfully converted in a 96% yield [28], but it too proved to be unsuccessful. Use of NaNH_2_ in ammonia was also tried but the conditions proved to be too harsh and decomposition ensued [43]. Another approach involved converting the chloro group using hydrazine followed by reduction. Initial attempts at reducing the hydrazine employed zinc in acetic acid, but this resulted in a complex mixture that could not be purified. Using titanium chloride (TiCl_3_) [44] proved to be successful, although there was evidence of some isopropylidene deprotected product(s) as well as protected products, thus treatment of the mixture with dilute TFA in THF gave the desired final product **1**.

Next, deaminated compound **10** was obtained from **8** using concentrated acetic acid at high temperature. Although this conversion also led to partial deprotection of the isopropylidene on the 2'- and 3'-hydroxyls, the protected pyridine **10** was the major product. Subsequent deprotection of the isopropylidene of **10** led to the fleximer inosine **3**.

### 2.2. Trypanosomiasis Screening

The three NpcA fleximers (**1**–**3**) were tested for trypanocidal activity against the laboratory *Trypanosoma brucei brucei* strain Lister 427, using a standard protocol based on the fluorescent format of 23 doubling dilutions, starting at 500 μM, in 96-well plates. All three fleximers tested displayed very similar activities against this strain, with EC_50_ values around 200 μM; in contrast, the control drug pentamidine displayed activity in the low nM range (Table 1), consistent with previous results [45,46].

**Table 1:** Trypanosomiasis results.

Compound	Average EC_50_ (µM)
**1**	216 ± 21
**2**	212 ± 31
**3**	287 ± 24
pentamidine	0.0044 ± 0.0001

EC_50_ = concentration of drug required to give a 50% response. Data are the average of three independent experiments and SEM.

**Figure 2:**
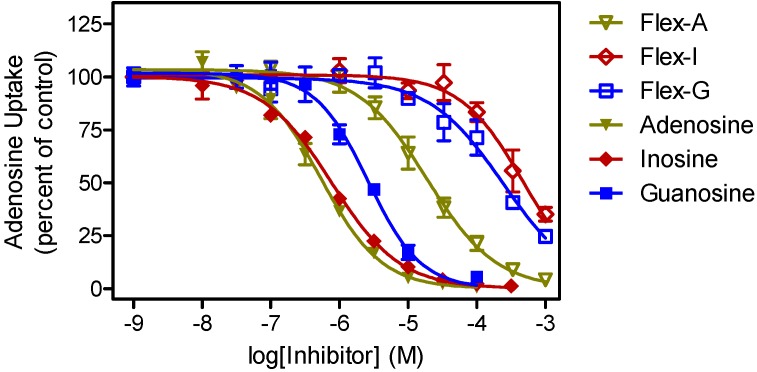
Transport of 0.1 µM [^3^H]-adenosine by *Trypanosoma brucei brucei* bloodstream form parasites.

We considered that the relatively low activity might be related to a lack of recognition of these molecules by the *T. brucei* nucleoside transporters. We therefore investigated whether fleximers in general display reduced uptake kinetics in these parasites, compared to their fixed-ring counterparts (Figure 2). Using the fleximers [21] most closely related to the original nucleoside substrates, it is clear from Table 2 that fleximers indeed show low affinity for the known *T. brucei* nucleoside transporters P1 and P2 [47].

**Table 2:** Comparison of affinity of purine nucleosides and corresponding fleximers for *T. brucei* transporters. 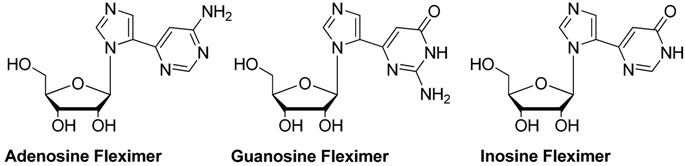

	P1 K_i_ (μM)			P2 K_i_ (µM)		
	Nucleoside ^1^	Fleximer	δ(ΔG^0^)	Nucleoside ^1^	Fleximer	δ(ΔG^0^)
Adenosine	0.36 ± 0.05	35 ± 11	11.4	0.91 ± 0.29	37 ± 3	9.2
Guanosine	1.8 ± 0.3	251 ± 75	12.2	>500	>500	
Inosine	0.44 ± 0.10	387 ± 30	16.8	>500	>500	

Data are the average inhibition constants (K_i_) and SEM of at least three independent experiments; Values for adenosine are Michaelis-Menten constants (K_m_). ^1^ values were taken from previous findings of De Koning [47] and included here for comparison; δ(ΔG^0^) is the difference in Gibbs free energy of interaction of the nucleoside and the fleximer with the transporter, given in kJ/mol.

Transport, mediated by the P1 nucleoside transporter, was measured in the presence or absence of various concentrations of nucleosides (filled symbols) or their corresponding fleximers (open symbols), in the presence of 100 µM adenine to block potential adenosine transport through the P2 transporter. Data shown are the average and SEM of triplicate determinations in a single experiment, representative of three independent experiments with essentially identical outcomes.

It is thus clear that the fleximers generally display about two orders of magnitude less affinity for the *T. brucei* nucleoside transporters than the corresponding nucleosides, limiting cellular uptake as there are no other nucleoside uptake mechanisms in these parasites than the P1 and P2 systems, although P1 consists of a cluster of multiple genes with slightly divergent sequences [48,49]. In addition, the truncated NpcA fleximers lack a 4'-hydroxymethyl group and an equivalent of the purine N3 residue, and both required for high affinity for P1 [50]. Moreover, the P2 transporter does not recognize any oxopurine nucleoside analogues [47]. The loss of approximately 10 kJ/mol in Gibbs free energy for the fleximer-transporter interaction may in part be due to the increased entropy in the orientation of the fleximer orientation in the binding pocket, as well as the slightly larger volume of the base. We thus conclude that the low effectiveness of the Npc fleximers is at least partially due to unfavorable interactions with the parasite’s nucleoside transporters. As important differences exist between nucleoside transporters of even closely related pathogenic parasites including *Trypanosoma congolense* [51] and *Leishmania* species [48], it would be worthwhile to follow this study with a wider screening of anti-parasite activity for a diverse panel of protozoa.

## 3. Experimental Section

### 3.1. General Information

All chemicals were obtained from commercial sources and used without further purification unless otherwise noted. Anhydrous DMF, MeOH, DMSO and toluene were purchased from Fisher Scientific (Pittsburgh, PA, USA). Anhydrous THF, acetone, CH_2_Cl_2_, CH_3_CN and ether were obtained using a solvent purification system (mBraun Labmaster 130, MBRAUN, Stratham, NH, USA). 3-Bromo-2-chloropyridine was obtained from Sigma-Aldrich (St. Louis, MO, USA). Melting points are uncorrected. NMR solvents were purchased from Cambridge Isotope Laboratories (Andover, MA, USA). All ^1^H- and ^13^C-NMR spectra were obtained on a JEOL ECX 400 MHz NMR, operated at 400 and 100 MHz respectively, and referenced to internal tetramethylsilane (TMS) at 0.0 ppm. The spin multiplicities are indicated by the symbols s (singlet), d (doublet), dd (doublet of doublets), t (triplet), q (quartet), m (multiplet), and b (broad). Reactions were monitored by thin-layer chromatography (TLC) using 0.25 mm Whatman Diamond silica gel 60-F_254_ precoated plates. Column chromatography was performed using silica gel (63–200 µm) from Dynamic Adsorptions Inc. (Norcross, GA, USA), and eluted with the indicated solvent system. Yields refer to chromatographically and spectroscopically (^1^H- and ^13^C-NMR) homogeneous materials. High resolution mass spectra were recorded at the Johns Hopkins Mass Spectrometry Facility (Baltimore, MD, USA) using fast atom bombardment for ionization.

### 3.2. Synthesis

*Preparation of (4R,5R)-4,5-O-isopropylidene-2-cyclopenten-1-ol* (**5**): (4*R*,5*R*)-4,5-*O*-isopropylidene-2-cyclopentenone **4** [35] (4.63 g, 0.03 mol) was dissolved in dry methanol (20 mL) at room temperature. CeCl_3_·7H_2_O was added to the reaction followed by the portionwise addition of NaBH_4_ (1.36 g, 0.04 mol). Once TLC analysis showed the complete disappearance **4**, the reaction was extracted into ethyl acetate (50 mL) and washed with water (10 mL). The organic layer was dried over MgSO_4_ and the solvent was removed under rotary evaporation. The crude oil was used in the following reaction without further purification.

*Preparation of (1′R,2′S,3′R)-1-[(2′,3′-O-isopropylidene)-4′-cyclopenten-1′-yl]-4,5-diiodoimidazole* (**6**): Thoroughly dried **5** (0.78 g, 0.05 mol) was dissolved in dry THF (100 mL) under N_2_. Ethylenebis(diphenylphosphine) (2.0 g, 0.05 mol) and 4,5-diiodoimidazole (3.2 g, 0.01 mol) added to the reaction, followed by the dropwise addition of diisopropyl azodicarboxylate. The reaction was allowed to stir for 48 h and then the solvent was removed using reduced pressure. The crude material was purified by silica gel column chromatography hexanes–ethyl acetate (2:1) to yield a yellow waxy solid (0.95 g, 0.02 mol, 41% yield). ^1^H-NMR (CDCl_3_): δ 1.32 (s, 3H), 1.45 (s, 3H), 4.44 (d, 1H, *J* = 5.5 Hz), 5.24 (d, 1H, *J* = 1.4 Hz), 5.31 (m, 1H), 5.90 (dt, 1H, *J* = 4.6, 5.9 Hz), 6.34 (dt, 1H, *J* = 1.8 Hz, 5.9 Hz), 7.38 (s, 1H). ^13^C-NMR (CDCl_3_): δ 25.9, 27.4, 70.8, 82.7, 84.2, 84.3, 97.1, 112.6, 128.9, 139.0, 139.4. HRMS calculated for C_11_H_12_I_2_N_2_O_2_ [M+H]^+^ 458.9066, found 458.9073.

*Preparation of (1′R,2′S,3′R)-1-[(2′,3′-O-isopropylidene)-4′-cyclopenten-1′-yl]-4-iodoimidazole* (**7**): Dried **6** (1.83 g, 0.004 mol) was dissolved in anhydrous THF under N_2_. The reaction was dropped to 0 °C, and ethyl magnesium bromide (3.0 M, 1.3 mL, 0.004 mol) was added dropwise to the reaction. After 1 h the reaction was quenched with saturated NH_4_Cl (5 mL) and then the solvent was removed. The crude mixture was dissolved in ethyl acetate (50 mL) and washed with water (20 mL) and then dried over MgSO_4_. The solvent was removed under reduced pressure and purified using silica gel chromatography petroleum ether–ethyl acetate (2:1) to yield a yellow oil (1.12 g, 0.003 mol, 85% yield). ^1^H-NMR (CDCl_3_): δ 1.28 (s, 3H), 1.40 (s, 3H), 4.44 (d, 1H, *J* = 5.5 Hz), 5.15 (d, 1H, *J* = 1.4 Hz), 5.30 (dq, 1H, *J* = 1.8, 5.5 Hz), 5.87 (dt, 1H, *J* = 0.9, 5.5 Hz), 6.22 (dt, 1H, *J* = 1.8, 5.9 Hz), 6.89 (d, 1H, *J* = 1.4 Hz), 7.34 (d, 1H, *J* = 1.4 Hz). ^13^C-NMR (CDCl_3_): δ 25.7, 27.3, 68.3, 82.7, 84.2, 84.9, 112.5, 123.2, 129.8, 137.5, 138.1. HRMS calculated for C_11_H_13_IN_2_O_2_ [M+H]^+^ 333.0100, found 333.0010.

*Preparation of 3-tributylstannyl-2-chloropyridine*: Commercially available 3-bromo-2-chloropyridine (0.30 g, 0.002 mol) was dissolved in anhydrous THF (20 mL) under N_2_. Ethyl magnesium bromide (3.0 M, 0.5 mL, 0.002 mol) was added dropwise at room temperature. The reaction was allowed to stir for 2 h, and then tributyltin chloride (0.42 mL, 0.002 mol) was added and the reaction was left to stir overnight. The reaction was concentrated *in vacuo* and then purified on silica gel chromatography hexanes-ethyl acetate (15:1) to yield a colorless oil (0.40 g, 0.001 mol, 64% yield). ^1^H-NMR (CDCl_3_): δ 1.13 (m, 5 H), 1.30 (m, 13H), 1.56 (m, 6 H), 1.65 (m, 3 H), 7.13 (dd, 1H, *J* = 4.6, 7.8 Hz), 7.67 (dd, 1H, *J* = 1.8, 4.6 Hz), 8.27 (dd, 1H, *J* = 1.8, 7.8 Hz). ^13^C-NMR (CDCl_3_): δ 26.9, 27.0, 27.3, 27.9, 28.9, 29.0, 29.1, 122.2, 139.5, 146.9, 147.1, 147.2, 149.6, 159.2.

*Preparation of (1′R,2′S,3′R)-3-[((2′,3′-O-isopropylidene)-4′-cyclopenten-1′-yl)-(imidazol-4-yl)]-2-chloropyridine* (**8**): Intermediate **7** (0.34 g, 0.001 mol) and 2-chloro-3-(tributylstannyl)pyridine (3.50 g, 0.007 mol) were dissolved in 1,4-dioxane under N_2_. Pd(PPh_3_)_4_ (0.05 g, 0.04 mmol) and CuBr (0.08 g, 0.5 mmol) were added to the reaction and the reaction and was refluxed at 120 °C for 12 h. The reaction was cooled and filtered through a pad of Celite. The filtrate was diluted in ethyl acetate (20 mL) and washed with a saturated solution of NH_4_Cl (20 mL), water (20 mL), brine (20 mL) and then dried over MgSO_4_. The organic solvent was removed under reduced pressure and the crude material was purified using 5% MeOH in CH_2_Cl_2_ to yield a yellow oil (0.23 g, 0.7 mmol, 71% yield). ^1^H-NMR (CDCl_3_): δ 1.36 (s, 3H), 1.48 (s, 3H), 4.58 (d, 1H, *J* = 5.5 Hz), 5.28 (d, 1H, *J* = 1.4 Hz), 5.40 (dt, 1H, *J* = 0.9, 4.6 Hz), 6.00 (dd, 1H, *J* = 1.2, 5.5 Hz), 6.32 (dt, 1H, *J* = 1.8, 5.5 Hz), 7.30 (dd, 1H, *J* = 4.6, 7.7 Hz), 7.55 (d, 1H, *J* = 1.4 Hz), 7.67 (d, 1H, *J* = 0.9 Hz), 8.26 (dd, 1H, *J* = 1.8, 4.6 Hz), 8.50 (dd, 1H, *J* = 1.8, 7.8 Hz). ^13^C-NMR (CDCl_3_): δ 25.7, 27.3, 68.5, 84.4, 85.1, 112.6, 118.5, 122.8, 129.4, 130.1, 132.1, 135.9, 136.9, 137.7, 137.9, 147.2. HRMS calculated for C_16_H_16_ClN_3_O_2_ [M+H ^35^Cl]^+^ 318.1009, [M+H ^37^Cl]^+^ 320.0980, found, 318.1001, 320.0976.

*Preparation of (1′R,2′S,3′R)-3-[((2′,3′-O-isopropylidene)-4′-cyclopenten-1′-yl)-(imidazol-4-yl)]-2-pyrimidone* (**10**): Analogue **9** (0.1 g, 0.3 mmol) was dissolved in concentrated acetic acid in a sealed glass tube and heated to 120 °C overnight. The acetic acid was evaporated and the crude material was extracted into ethyl acetate. Silica gel chromatography using ethyl acetate-acetone-methanol (6:1:1) yielded an off white solid (0.05 g, 0.2 mmol, 56% yield). ^1^H-NMR (DMSO-*d_6_*): δ 1.24 (s, 3H), 1.32 (s, 3H), 4.41 (d, 1H, *J* = 6.9 Hz), 5.05 (d, 1H, *J* = 4.6 Hz), 5.98 (d, 1H, *J* = 5.9 Hz), 6.12 (dt, 1H, *J* = 2.3, 5.9 Hz), 6.26 (t, 1H, *J* = 6.4 Hz), 7.24 (d, 1H, *J* = 4.6 Hz), 7.65 (d, 1H, *J* = 0.9 Hz), 7.79 (d, 1H, *J* = 1.4 Hz), 8.07 (dd, 1H, *J* = 1.8, 7.3 Hz), 11.78 (bs, 1H). ^13^C-NMR (DMSO-*d_6_*): δ 25.5, 27.3, 67.4, 73.0, 78.9, 105.8, 117.6, 125.1, 131.0, 131.6, 132.9, 133.9, 136.7, 136.8, 136.9, 160.7. HRMS calculated for C_16_H_17_N_3_O_3_ [M+H]^+^ 300.1348, found 300.1342.

*Preparation of (1′R,2′S,3′R)-3-[(2ʹ,3ʹ-Dihydroxy)-4ʹ-cyclopenten-1ʹ-yl]-(imidazol-4-yl)-2-amino-pyridine* (**1**): Compound **8** (80 mg, 0.25 mmol) was refluxed in hydrazine (2 mL) for 1 h. The solvent was removed under reduced pressure and the residue dissolved in THF (5 mL). Titanium (III) chloride (1.7 mmol, 0.5 mL, 20% in 3% HCl) was neutralized using NaOH (0.5 mL, 20%), and 0.6 mL of the solution was added dropwise to the reaction. The mixture was refluxed at 70 °C for 4 h, cooled to room temperature, and brought to pH > 10 using NaOH (20%) while cooling in an ice-bath. The solvents were removed under vacuum, and the product was extracted with CH_2_Cl_2_ (10 mL × 5). The organic layer was dried over MgSO_4_. The solvents were evaporated, and the crude material, 3-[((2', 3'-*O*-isopropylidene)-4'-cyclopenten-1'-yl)-imidazol-4-yl]-2-aminopyridine (**9**), was used directly in preparation of **1**. Crude **9** was dissolved in THF (5 mL) and TFA:H_2_O (1 mL:1 mL) was added dropwise. This was allowed to stir overnight at room temperature. The solvent was evaporated and co-evaporated with ethanol (3 × 5 mL) to yield an off-white solid (0.03 g, 0.1 mmol, 46% yield over 2 steps). ^1^H-NMR (DMSO-*d_6_*): δ 3.93 (m, 1H), 4.45 (m, 1H), 4.96 (m, 1H), 5.08 (m, 1H), 5.14 (d, 1H, *J* = 6.9 Hz), 5.96 (dd, 1H, *J* = 1.4, 6.4 Hz), 6.11 (dt, 1H, *J* = 2.3, 6.4 Hz), 6.54 (dd, 1H, *J* = 5.0, 7.3 Hz), 6.95 (bs, 2H), 7.59 (d, 1H, *J* = 0.9 Hz), 7.70 (dd, 1H, *J* = 1.8, 7.4 Hz), 7.78 (d, 1H, *J* = 1.0 Hz), 7.81 (m, 1H). ^13^C-NMR (DMSO-*d_6_*): δ 67.6, 73.1, 78.6, 112.2, 112.5, 115.1, 132.9, 133.7, 136.2, 136.9, 140.1, 146.5, 156.5 HRMS calculated for C_13_H_14_N_4_O_2_ [M+H]^+^ 259.1195, found 259.1193.

*Preparation of (1′R,2′S,3′R)-3-[(2ʹ,3ʹ-Dihydroxy)-4ʹ-cyclopenten-1ʹ-yl]-(imidazol-4-yl)-2-chloro-pyridine* (**2**): Intermediate **8** (0.16 g, 0.5 mmol) was dissolved in THF (5 mL) and TFA:H_2_O (1 mL:1 mL) was added dropwise. This was allowed to stir overnight at room temperature. The solvent was evaporated and co-evaporated with ethanol (3 × 5 mL). Column chromatography in 10% MeOH in CH_3_CN returned an off-white solid (0.12 g, 0.4 mmol, 86% yield). ^1^H-NMR (DMSO-*d_6_*): δ 3.90 (m, 1H), 4.42 (m, 1H), 4.98 (m, 1H), 5.08 (m, 1H), 5.17 (d, 1H, *J* = 6.4 Hz), 6.00 (dd, 1H, *J* = 1.4, 5.9 Hz), 6.11 (dt, 1H, *J* = 2.3, 5.9 Hz), 7.43 (dd, 1H, *J* = 4.6, 7.8 Hz), 7.77 (s, 1H), 7.80 (s, 1H), 8.22 (dd, 1H, *J* = 2.3, 4.6 Hz), 7.81 (dd, 1H, *J* = 1.8, 7.8 Hz). ^13^C-NMR (DMSO-*d_6_*): δ 67.6, 73.0, 78.9, 119.0, 123.8, 129.9, 132.7, 135.4, 137.1, 137.6, 137.9, 146.5, 147.5. HRMS calculated for C_13_H_12_ClN_3_O_2_ [M+H ^35^Cl]^+^ 278.0696, [M+H ^37^Cl]^+^ 280.0667, found, 278.0689, 280.0668.

*Preparation of (1′R,2′S,3′R)-3-[(2ʹ,3ʹ-Dihydroxy)-4ʹ-cyclopenten-1ʹ-yl]-imidazol-4-yl)-2-hydroxypyridine* (**3**): Intermediate **10** (0.08 g, 0.3 mmol) was dissolved in THF (5 mL) and TFA:H_2_O (1 mL:1 mL) was added dropwise. This was allowed to stir overnight at room temperature. The solvent was evaporated and co-evaporated with ethanol (3 × 5 mL). Column chromatography in ethyl acetate–acetone–methanol–water (6:1:1:0.5) produced an off-white solid (0.03 g, 0.1 mmol, 43% yield). ^1^H-NMR (DMSO-*d_6_*): δ 3.82 (d, 1H, *J* = 4.6 Hz), 4.43 (bs, 1H), 4.95 (m, 1H), 5.05 (m, 1H), 5.11 (d, 1H, *J* = 7.4 Hz), 5.97 (dd, 1H, *J* = 1.4, 5.9 Hz), 6.12 (dt, 1H, *J* = 2.8, 5.9 Hz), 6.29 (t, 1H, *J* = 6.9 Hz), 7.23 (d, 1H, *J* = 4.6 Hz), 7.65 (d, 1H, *J* = 0.9 Hz), 7.80 (d, 1H, *J* = 1.4), 8.08 (dd, 1H, *J* = 2.3, 7.3 Hz), 11.73 (bs, 1H). ^13^C-NMR (DMSO-*d_6_*): δ 67.6, 73.1, 78.6, 112.2, 112.5, 115.1, 132.9, 133.7, 136.2, 136.9, 140.1, 146.5, 156.5 HRMS calculated for C_13_H_13_N_3_O_3_ [M+H]^+^ 260.1035, found 260.1033.

### 3.3. Anti-Trypanosome Activity

*In vitro* activity against *Trypanosoma brucei* was determined using the Alamar blue (resazurin) assay for cell viability exactly as described [52]. Briefly, serial dilutions of test compounds were made in 96-well plates by serial passage of 100 μL of test compound (usually at 2 mM) to 100 μL of HMI9 medium containing 10% fetal bovine serum (Invitrogen), using 2 rows, with the negative control values obtained from wells with 100 μL of medium without test compound. Serial dilutions with pentamidine isethionate (Sigma) were used as positive control. To each well, 100 μL of medium, containing 10^4^ culture-adapted bloodstream *T. b. brucei* (strain Lister 427), was added and the plates were incubated at 37 °C for 48 h after which 20 μL 5 mM resazurin solution was added. Following a further incubation of 24 h at 37 °C, fluorescence was determined in a FLUOstar OPTIMA (BMG Labtech, Aylesbury, UK) fluorimeter with excitation and emission filters at 544 nm and 620 nm, respectively. EC_50_ values (the effective concentration reducing specific fluorescence by 50%) were calculated by nonlinear regression using the Prism 5 software package (GraphPad, La Jolla, CA, USA).

### 3.4. Transport Assays

Transport assays with bloodstream forms of *T. b. brucei* were performed exactly as described previously [53,54]. Briefly, transport was initiated by the addition of 100 µL of *T. b. brucei* bloodstream forms (10^7^ cells/mL in assay buffer [53]) to 100 µL of [2,8,5'-^3^H]-adenosine (PerkinElmer, Waltham, MA, USA; 54.4 Ci/mmol) pre-mixed with up to 1 mM of test inhibitor in assay buffer. After exactly 10 s the mixture was centrifuged through an oil layer in a microfuge (13,000× *g*) and the microfuge tubes were flash-frozen in liquid nitrogen. Pellets were cut off and collected in scintillation tubes; after solubilisation in 2% SDS and addition of scintillation fluid, radioactivity was determined in a liquid scintillation counter. Inhibition data were fitted to a sigmoidal curve with variable slope (GraphPad Prism 5.0), allowing for the determination of EC_50_ values, from which inhibition constants (K_i_) were calculated using the Cheng-Prusoff equation, and Gibbs Free Energy using ΔG^0^ = −RTln (K_i_), as described [52].

## 4. Conclusions

The strategy of the work presented herein was to potentially synthesize new and more potent inhibitors of SAHases, thereby disrupting mRNA capping in protozoa as a strategy towards new antiparasitic therapeutics. To this end, characteristics of known SAHase inhibitors such as neplanocin and Aristeromycin were combined, and the nucleoside analogue was given enhanced flexibility using the “fleximer” approach, and added specificity by omitting the N3 equivalent nitrogen residue in the pyrimidine half of the fleximer base group. In addition, the 4'-CH_2_OH moiety was omitted to reduce general cytotoxicity [10,14]. The data, however, show that the resulting 3-deazaneplanocin fleximers (**1**–**3**) displayed only moderate activity in a standardized anti-protozoal test, against *Trypanosoma brucei*, despite the possibility of this species being vulnerable to inhibition of SAHase [55].

We have previously shown that the trypanocidal action of nucleoside and nucleobase analogues is either enabled or limited by their rate of uptake by specific transport proteins [52,53,54,56,57], and therefore investigated the effect of the fleximer modification on nucleoside transport. We found that the introduction of this modification of the purine ring reduces affinity, and thus presumably translocation rates, for both of the transport systems expressed in bloodstream *T. brucei*, and conclude that the lack of suitable transporters for these molecules causes (or at least contributes to) the observed lack of trypanocidal potency. However, we have also shown that purine transporters in other protozoan parasites, e.g., *Toxoplasma gondii* [58], *Plasmodium falciparum* [59], *Leishmania donovani* [48], and *Trichomonas vaginalis* (Natto and De Koning, unpublished data) all have very different substrate-specificity characteristics. Further studies with additional parasites, and the optimization of the inhibitors for enhanced uptake by the parasites, are in progress.
